# Off-label medicine use/prescribing controversies through patients' eyes/“rights”: the example of povidone-iodine enemas/suppositories for “terminal” pouchitis

**DOI:** 10.11604/pamj.2019.33.155.19222

**Published:** 2019-07-01

**Authors:** Constantine Davanas

**Affiliations:** 1 Ministry of Transport, Amphanon Str. No.17, Saint Stephen 38500, Greece

**Keywords:** Off-label medicine use/prescribing, povidone iodine, pouchitis, patients´ rights

## To the editors of the Pan African Medical Journal

There is an abundance of literature addressing the everlasting legal/ethical/moral controversies about the off-label use/prescribing of medicines and the need to streamline the practice [1,2]. The issue is visited herein from the patient's viewpoint, via a particular example and a suggestion is put forth. Pouchitis is the most frequent long-term complication for inflammatory bowel disease (IBD) patients who have undergone ileal pouch-anal anastomosis [3]. While antibiotics are routinely effective against the inflammation of the constructed pouch (“pouchitis” classified as IBD), indicating that a pathogen might be involved, there is a significant proportion of patients who end up having chronic antibiotic refractory pouchitis, for whom the future is bleak (if they are to avoid pouch excision and permanent ileostomy): mesalazine and immunosuppressants are prescribed, without a firm basis of clinical trials, at severe infection risks (for the latter) and frequently at very high costs (i.e. for tumor necrosis factor alpha antagonists), with dubious outcomes. Thus, a patient facing unremitting disease/pouchitis, with major complications and medication side-effects, is often willing to risk off-label medicines never used before for pouchitis, provided he/she can find a physician willing to prescribe (*“quasi-experimentally”*) and accept the legitimacy of his/her choice.

Povidone iodine (betadine) has never been included in the armamentarium against pouchitis in spite of its excellent record as a topical antiseptic lethal to bacteria, fungi, viruses, protozoa. Also, despite of it being widely used (both externally and internally) for over five decades, with minimal toxicity or side-effects (especially suitable for mucous surfaces at the right/low concentration) and without development of tolerance (not to mention its ultra-low cost, a major factor for developing countries) [4-7]. Examples abound: preparation (localized/swab/gauze, enema or suppository) for transrectal prostate biopsies, where its use decreases complications/infections by ~1 order of magnitude; intraperitoneal irrigation in the face of infection; pre-/post- surgery external/internal usage; minor/major external wound treatment; vaginitis (endogenital) treatment (literally a multi-million cohort of women would attest to this); multi-year rectal suppository (complementary) treatment for radiation proctitis/fistulae; accidental oral administration [4-9]. Hence, why not take a shot at povidone iodine enemas/suppositories? After all, a typical two inch (“diameter”) by five inch (“length”) pouch is by definition a “topical” application site. It goes without saying that such a povidone iodine treatment of pouchitis has to proceed hand in hand with regular monitoring of the patient. Possible legal repercussions and the fact that it has never been tried before (*“quasi-experimental”*), makes doctors reluctant to prescribe, despite the absence of counter-argumentation (in own clinical experience or literature) and the willingness (and *right*) of the patient to assume full responsibility and risks.

A possible way out of the deadlock: unstigmatize (“streamline”) the “quasi-experimental” off-label use/prescription when the drug is included in the World Health Organization's “list of essential medicines”. That could offer patients a last resort; generalizing the presented example: perhaps (last resort) not only for pouchitis-stricken people, but for IBD sufferers in general, because if there is a role for antibiotics in IBD (besides pouchitis) [10] and thus pathogen involvement, there might be a role for povidone iodine (which is included in the “list of essential medicines”) as well, especially when (only) the lower large intestine is inflamed. First time off-label use of a drug could be deemed devoid of legal repercussions for the prescribing doctors when the drug appears in the World Health Organization's “list of essential medicines” and the patient insists (regarding it as his/her “right”) on assuming all responsibility/risks, as in the presented example of a patient willing to use povidone iodine enemas/suppositories for “terminal” pouchitis.

## Competing interests

The author declares no competing interests.
